# Mitochondrial DNA Heteroplasmy as an Informational Reservoir Dynamically Linked to Metabolic and Immunological Processes Associated with COVID-19 Neurological Disorders

**DOI:** 10.1007/s10571-021-01117-z

**Published:** 2021-06-12

**Authors:** George B. Stefano, Richard M. Kream

**Affiliations:** Center for Cognitive and Molecular Neuroscience, First Faculty of Medicine, Charles University in Prague, Prague, Czech Republic

**Keywords:** Mitochondrial DNA, Immune response, Central nervous system, Gut microbiome, SARS-CoV-2, COVID-19

## Abstract

Mitochondrial DNA (mtDNA) heteroplasmy is the dynamically determined co-expression of wild type (WT) inherited polymorphisms and collective time-dependent somatic mutations within individual mtDNA genomes. The temporal expression and distribution of cell-specific and tissue-specific mtDNA heteroplasmy in healthy individuals may be functionally associated with intracellular mitochondrial signaling pathways and nuclear DNA gene expression. The maintenance of endogenously regulated tissue-specific copy numbers of heteroplasmic mtDNA may represent a sensitive biomarker of homeostasis of mitochondrial dynamics, metabolic integrity, and immune competence. Myeloid cells, monocytes, macrophages, and antigen-presenting dendritic cells undergo programmed changes in mitochondrial metabolism according to innate and adaptive immunological processes. In the central nervous system (CNS), the polarization of activated microglial cells is dependent on strategically programmed changes in mitochondrial function. Therefore, variations in heteroplasmic mtDNA copy numbers may have functional consequences in metabolically competent mitochondria in innate and adaptive immune processes involving the CNS. Recently, altered mitochondrial function has been demonstrated in the progression of coronavirus disease 2019 (COVID-19) due to severe acute respiratory syndrome coronavirus 2 (SARS-CoV-2) infection. Accordingly, our review is organized to present convergent lines of empirical evidence that potentially link expression of mtDNA heteroplasmy by functionally interactive CNS cell types to the extent and severity of acute and chronic post-COVID-19 neurological disorders.

## Introduction

Human mitochondrial DNA (mtDNA) consists of a circular genome of 16,569 base pairs (bp) that contains 13 protein-encoding genes traceable to primordial maternal haplogroups (Anderson et al. 1981; Taanman 1999). The subunits ND1-3, ND4, ND4-6 of Complex I, cytochrome b, subunits CO1-3 of Complex IV, and subunits A6 and A8 of Complex V (ATP synthase) expressed from mtDNA represent essential components of the electron transport chain. Nuclear diploid gene sequences encode the vast majority of cognate protein subunits, accessory proteins, and assembly-related protein chaperones. Complex regulatory factors driving mtDNA gene expression in response to physiological or pathological changes in cellular energy demands are reciprocally dynamic, and they functionally extend to a multiplicity of nuclear target genes (Quiros et al. 2016). Within this mechanistic context, heteroplasmy of mtDNA specifically refers to dynamically determined co-expression of wild type (WT) inherited polymorphisms and collective time-dependent somatic mutations within individual mtDNA genomes (see Stefano et al. 2017).

The total number of mitochondria and mtDNA copy numbers vary by at least three orders of magnitude within the individual cell and tissue types (Allen 2015; He et al. 2010; Jacobi et al. 2001; Stefano et al. 2017). The patterns of expression and distribution of heteroplasmic mtDNA may represent a regulatory mechanism functionally linked to the maintenance of ATP levels in response to physiological demand (Allen 2015; He et al. 2010; Jacobi et al 2001; Stefano et al. 2017). The review presents interrelated topics that integrate recently elucidated mechanistic aspects of mtDNA heteroplasmy with special reference to mitochondrial associated immune processes across multiple cell types that include myeloid cells, monocytes, macrophages, and antigen-presenting dendritic cells. Our overall goal is to critically discuss and integrate convergent lines of empirical evidence that potentially link expression of mtDNA heteroplasmy by functionally interactive CNS cell types to the extent and severity of acute and chronic post-COVID-19 neurological disorders.

## Functional Relevance of mtDNA Heteroplasmy to Human Health and Disease

Previously we have presented a functionally-oriented, multifaceted review of mitochondrial heteroplasmy from cellular, physiological, and pathophysiological perspectives (Stefano et al. 2017). Many studies have focused on identifying the potential etiological roles of heritable mutations in protein-encoding genes and mtDNA genes with sporadic somatic mutations (Davila and Zamorano 2013; He et al. 2010; Stefano et al. 2017). These mutations have been identified in congenital cardiomyopathies, neurological diseases, and human malignancy (Davila and Zamorano 2013; He et al. 2010; Stefano et al. 2017). The maintenance of adequate cell energy within dynamically changing physiological environments may be compromised following the loss of function mutations in protein-encoding mtDNA genes (Aryaman et al. 2019; Hoitzing et al. 2019). Accordingly, we have surmised that cell-specific and tissue-specific heteroplasmic mtDNA expression in healthy individuals is functionally associated with bidirectional intracellular communication involving mitochondrial signaling pathways and nuclear DNA gene expression (Stefano et al. 2017).

Stochastic modeling algorithms have been developed to provide theoretical frameworks that functionally link variability in cellular distributions of heteroplasmic mtDNA within mitochondrial networks (Aryaman et al. 2019; Hoitzing et al. 2019). These analyses suggest that complex regulatory factors underlying fusion and fission ratios are functionally associated with state-dependent variance in the mean distribution of heteroplasmic mtDNA copy numbers (Aryaman et al. 2019; Hoitzing et al. 2019). Accordingly, empirically determined tissue-specific variances in the rates of formation of heteroplasmic mtDNA distributions may represent biomarkers of optimized mitochondrial dynamics (Aryaman et al. 2019; Hoitzing et al. 2019). Within a clinical context, prospective studies on the homeostatic regulatory factors that underlie the observed intrinsic differences in cell-specific and organ-specific distribution of heteroplasmic mtDNA may result in novel treatment approaches for mitochondrial disease or enhanced quality of life for aging populations.

Theoretical modeling studies are complemented and empirically validated by in vivo analyses of engineered heteroplasmic mouse strains with nonpathological mtDNA polymorphisms (Lechuga-Vieco et al. 2020). In heteroplasmic mice, integrative analysis showed non-random segregation of mtDNA copy numbers in all tissues studied (Lechuga-Vieco et al. 2020). The authors concluded that mtDNA heteroplasmy reflected an intracellular sorting process based on organelle selection that was critically dependent on the functional coupling of the rate of mitochondrial oxidative phosphorylation to cellular metabolic requirements (Lechuga-Vieco et al. 2020).

## Functional Relevance of mtDNA Heteroplasmy to Mitochondrial Transfer Mechanisms

In light of the above, the combination of stochastic modeling and complementary studies of heteroplasmic mouse strains with nonpathological mtDNA polymorphisms has our increased understanding of the potential depth of biological roles linked to mtDNA heteroplasmy (Aryaman et al. 2019; Hoitzing et al. 2019; Lechuga-Vieco et al. 2020). Within the last decade, key studies have supported the physiological importance of state-dependent transfer of functional mitochondria from healthy donor cells to metabolically compromised cells (Stefano and Kream 2016). Operationally, advantageous mitochondrial transfer indicates a superimposition of functionally competent heteroplasmic mtDNA copy numbers within the cell to restore mitochondrial dynamics within multiple organ systems (Jackson et al. 2016; Kitani et al. 2014; McCoy-Simandle et al. 2016; Tatsuta et al. 2014; Wang and Gerdes 2015). In cell-specific or organ-specific systems, intercellular transfer of functional mitochondria involves synergistic mechanisms involving tunneling nanotubes and/or cellular-derived exosomes as delivery systems (Jackson et al. 2016; Kitani et al. 2014; McCoy-Simandle et al. 2016; Tatsuta et al. 2014; Wang and Gerdes 2015). We contend that the summated effects of mass transfer of physiologically competent mitochondria in response to physiological demands may involve restorative transcriptional and translational processes mediated by functional heteroplasmic mtDNA copy numbers within cytoplasmic microdomains.

From translational perspectives, continuation of integrated biochemical and molecular studies designed to critically probe complex mechanisms of intercellular transport of functional mitochondria are required to critically validate the safety and efficacy of using functionally viable mitochondria as a novel therapeutic approach to restore cellular and organ function in disease and healthy aging (Stefano and Kream 2016; McCully et al. 2016). Recent advances in the use of selective CRISPR gene editing and functional reversal of heritable mtDNA mutations in mitochondrial diseases have supported the possibility of using these molecular bioengineering technologies (Mok et al. 2020). Accordingly, future therapeutic usage of mitochondrial replacement therapy employing highly purified preparations of autologous mitochondria previously engineered to provide optimal oxidative phosphorylation and ATP production represents a potentially novel approach for treating heritable mitochondrial diseases and well-studied metabolic disorders such as Type II diabetes. Similar considerations may apply to the potential future use of mitochondrial replacement therapy with selectively engineered mtDNA copy numbers for targeted therapy or personalized medicine in genetic, infectious, and degenerative diseases (Pacheu-Grau et al. 2013). In these therapeutic applications, tissue-specific and organ-specific distribution of heteroplasmic mtDNA copy numbers may provide quality control parameters for the successful application of mitochondrial replacement therapy strategies.

Finally, intact cell-free mitochondria with normal energy production have been identified in healthy human blood samples (Al Amir Dache et al. 2020). In this study, the authors estimated that between 200,000 and 3,700,000 functional mitochondria per ml of extracted plasma were present (Al Amir Dache et al. 2020). They proposed that circulating cell-free functional mitochondria were representative of a novel class of signaling organelles involved in complex regulatory activities and intercellular communication (Al Amir Dache et al. 2020). However, it remains to be confirmed whether blood-borne functional mitochondria serve as sentinels for maintaining homeostatic metabolic activities and a reserve for essential cellular functions.

## Modulation of Interactive Immune Responses by mtDNA Heteroplasmy

A major goal of our research has been to investigate the functional roles of key mitochondrial regulatory molecules in the pathophysiology of acute and chronic inflammation, with special reference to altered expression of proinflammatory and anti-inflammatory cytokines and their protein receptor complexes (Esch and Stefano 2002; Esch et al. 2020; Stefano et al. 2015). A physiological state of proinflammation is a key autoregulatory mechanism that maintains immune surveillance of infective microorganisms, including viruses (Esch and Stefano 2002; Esch et al. 2020). In support of this contention, myeloblast-derived immune cells, monocytes, macrophages, and dendritic antigen-presenting cells undergo distinct programmed metabolic changes in mitochondrial bioenergetics in innate and adaptive immunological processes (Angajala et al. 2018; Zuo and Wan 2019). Polarized M1 macrophages utilize uncoupled mitochondrial oxidative phosphorylation functionally coupled to a disrupted cell cycle to maintain a proinflammatory phenotype (Angajala et al. 2018; Zuo and Wan 2019). M2 macrophages retain concerted mitochondrial systems to produce anti-inflammatory responses (Angajala et al. 2018; Zuo and Wan 2019). Immunologically-driven mitochondrial processes are associated with morphological changes and altered ratios of fusion and fission linked to mitophagy rates and junctional coupling to the endoplasmic reticulum (Angajala et al. 2018; Zuo and Wan 2019). The potential regulatory roles of heteroplasmic mtDNA copy numbers to successfully drive these integrative cellular processes have been discussed above ((Aryaman et al. 2019; Hoitzing et al. 2019; Lechuga-Vieco et al. 2020).

Recent studies have observed multiple proinflammatory effects from extracellular transport and uptake of mitochondrial degradation products from sites of cell and tissue damage or high intracellular mitophagy rates (Pollara et al. 2018; Zhu et al. 2018; Nagakawa et al. 2020). Diverse mitochondrial degradation products have been qualitatively termed mitochondrial damage-associated molecular patterns (mtDAMPs) and include N-formylated peptide fragments derived from mitochondrial proteins, intact full-length mtDNA copy numbers, mtDNA fragments, and small organic molecules such as ATP and cardiolipin that are functionally linked to ongoing innate immune responses to pathogens (Al Amir Dache et al. 2020; Esch et al. 2020; Chandel 2014; Tiku et al. 2020; West et al. 2011). In healthy individuals, ongoing cell turnover may result in a basal level of blood-borne mtDAMPs and circulating mitochondria to maintain a constitutive proinflammatory state that is significantly enhanced after immunological challenges by infectious pathogens. Interestingly, intact mitochondria released from apoptotic or necrotic dying cells have been observed to directly mediate proinflammatory processes with downstream effects on adaptive immune responses that may promote long term pathophysiological effects under chronic conditions (Pollara et al. 2018; Zhu et al. 2018; Nagakawa et al. 2020).

In light of the above, a recent study has provided empirical evidence in support of novel signaling activities of blood-borne mitochondria to mediate innate immune cell-to-cell communication (Song et al. 2020). Intact and cell-free mitochondria from human plasma were shown to present membrane-associated, immunologically active proteins, including CD270 and programmed cell death-ligand 1 (PD-L1) (Song et al. 2020). Furthermore, intact mitochondria from human plasma and immunologically active surface proteins were associated with upregulation of activated CD4 + T-cells and CD8 + T-cells and reduced concentrations of proinflammatory cytokines (Song et al. 2020). Additional studies are warranted to critically evaluate the potential regulatory roles of differences in the distribution of heteroplasmic mtDNA copy numbers within blood-borne metabolically competent mitochondria in the partial mediation of innate immune responses.

## Modulation of the Immune Response Involving the Gut-Brain Axis by Heteroplasmic mtDNA

Communication between intrinsic luminal and immune cell populations and commensal strains of gut microbiota maintain homeostatic innate and adaptive immune processes within the gastrointestinal (GI) tract (Stefano et al. 2018). Recent studies have identified a pivotal role of host cell mitochondria and immune signaling to maintain optimal diversity and functional integrity of gut bacteria commensal strains (Hirose et al. 2017; Saint-Georges-Chaumet and Edeas 2016; Yardeni et al. 2019). Mitochondrial oxidative phosphorylation is functionally coupled to a disrupted mitochondrial tricarboxylic acid cycle (TCA) cycle and is associated with a proinflammatory phenotype in enteric immune cells, mediated by reactive oxygen species (ROS) (Saint-Georges-Chaumet and Edeas 2016; Yardeni et al. 2019). Enteric microglia found in Auerbach's plexus and Meisner’s plexus of the enteric nervous system are key players in maintaining innate immunity in the GI tract (Verheijden et al. 2015). The sustained release of proinflammatory mediators within the gut converts enteric microglia into active immune cells with an M1 macrophage-like phenotype (Sonetti et al. 1994; Stefano et al. 1994).

As previously discussed, activation of innate immune responses to infection by pathogens is dependent on functionally competent mitochondria. Within the enteric nervous system and GI tract, a complementary array of normative mitochondrial functions is adversely affected during infection by bacterial pathogens (Lobet et al. 2015). Repeated exposure to outer membrane vesicles from Gram-negative bacteria may induce mitochondrial dysfunction and apoptosis functionally linked to the activation and sustained release of interleukin-1β to enhance proinflammatory processes in the GI tract (Deo et al. 2020). Recently reported studies from a mouse model evaluated the downstream effects of selective single nucleotide mutations in mitochondrial respiratory complex protein genes expressed by mtDNA open reading frames (ORFs) on changes in mitochondrial function and immune processes and differences in populations of gut microbiota (Hirose et al. 2017, 2019; Schilf et al. 2021). In sum, these data gleaned from several complementary studies on heteroplasmic mouse strains strongly suggest an important regulatory role of mtDNA heteroplasmy within mitochondria of luminal and immune gut cells to maintain mitochondrial bioenergy homeostasis and innate immune signaling processes.

Bidirectional gut-brain signaling pathways are reciprocally regulated by metabolic and immune processes of the host GI cell mitochondria (Diaz Heijtz et al. 2011; Zhou and Foster 2015). Conversely, dysbiosis of major strains of commensal gut microbiota has been functionally associated with degenerative CNS disorders that include Parkinson’s disease (Bedarf et al. 2017; Devos et al. 2013; Forsyth et al. 2011; Malkki 2017; Mulak and Bonaz 2015; Rietdijk et al. 2017; Scheperjans et al. 2015), Alzheimer's disease, Huntington's disease, and amyotrophic lateral sclerosis (ALS) (Main and Minter 2017; Tremlett et al. 2017). Several comorbid pathophysiological events have been shown to precede classic motor symptoms in Parkinson’s disease and include dysbiosis of the gut microbiota, loss of intrinsic enteric nervous system and lymphoid coupling, and progressive colonic inflammation (Bodea et al. 2014; Braak et al. 2003; Dobbs et al. 1999; Forsyth et al. 2011; Kosikowska et al. 2016; Lebouvier et al. 2009; Rietdijk et al. 2017; Villaran et al. 2010). It is therefore possible that the pathophysiology of Parkinson’s disease involves the slow progression of degenerative changes in the gut transmitted by peripheral nerves and humoral regulatory loops to the CNS with degeneration of the nigrostriatal CNS pathway (Braak and Del Tredici 2017; Braak et al. 2003; Rietdijk et al. 2017). More recently, the possible functional roles of heritable polymorphisms in mtDNA open reading frames (ORFs) have been investigated as a source of mitochondrial dysfunction in Parkinson’s disease-associated neurodegenerative processes (Müller-Nedebock et al. 2021).

## Modulation of Mitochondrial, Metabolic, and Immune Responses in COVID-19 Neurological Disorders by Heteroplasmic mtDNA

A review of complementary in vivo, in vitro and neuropathological studies has highlighted convergent and potentially synergistic strategies used by SARS-CoV-2 for neuro-invasion, infection, and replication within CNS neurons and glia with subsequent cellular and tissue damage linked to multifaceted neurological dysfunction (Crunfli et al. 2020; Matschke et al. 2020; Song et al. 2021). Song and coworkers utilized a three pronged self-validating approach to evaluate the mechanistic capacity of SARS-CoV-2 to infect the CNS via in vitro usage of human brain organoids, in vivo modeling employing transgenic mice overexpressing human ACE2, and analysis of human autopsy specimens from patients who died from COVID-19-associated pathologies. Overall, the authors provided evidence for the neuroinvasive capacity of SARS-CoV-2 in an apparent ACE2-dependent mechanism for cellular entry and replication. Furthermore, they empirically demonstrated that CNS structures are sites for high replicative potential for SARS-CoV-2 and that SARS-CoV-2 infection subsequently engenders significant neuronal death in the in vitro human brain organoids model. Examination of dying cells in the organoid cultures indicated that neuronal death did not directly colocalize with viral infection, thereby suggesting that infected cells are capable of engendering regions of hypoxia within microdomains that potentially lead to tissue damage due to metabolic insufficiencies. Importantly, these observations are line with current critical thinking that links COVID-19 progression with successful takeover or hijacking of essential mitochondrial processes, including oxidative phosphorylation and ATP production (Burtscher et al. 2020; Stefano et al. 2020, 2021; Wang et al. 2020). Accordingly, successful SARS-CoV-2 infection is proposed to be functionally associated with a functional override of antiviral innate immune responses associated with multiple mitochondrial processes (Burtscher et al. 2020; Shenoy, 2020).

As discussed above, enteric microglia found in Auerbach's plexus and Meisner’s plexus of the enteric nervous system are key players in maintaining innate immunity in the GI tract (Verheijden et al. 2015) and sustained release of proinflammatory mediators within the gut converts enteric microglia into active immune cells with an M1 macrophage-like phenotype (Sonetti et al. 1994; Stefano et al. 1994). In similar fashion, neuroinflammatory conditions within the CNS induce brain microglia to undergo mitochondrial-dependent metabolic reprogramming from oxidative glycolysis towards aerobic glycolysis (Bernier et al. 2020; Lynch 2020). Aberrant neuroimmune responses engendered by polarized microglia that are functionally linked to widespread metabolic dysregulation appear to represent common components of neurodegenerative diseases, COVID-19 neurological disorders and coordinate damage to CNS neurons and astrocytes (Crunfli et al. 2020; Matschke et al. 2020; Song et al. 2021). Interestingly, extracellular release of functionally competent mitochondria from astrocytes coupled to uptake by damaged neurons utilizing an in vivo rodent stroke model has been observed to promote enhanced cell survival signaling and neurological outcome (Hayakawa et al. 2016). As discussed above, additional studies are warranted to critically evaluate the potential regulatory roles of differences in the distribution of heteroplasmic mtDNA copy numbers within metabolically competent mitochondria in damaged CNS neurons and glia subsequent to COVID-19 infection.

A high resolution predictive index of the extent of SARS-CoV-2 neuro-invasion may be obtained from a computational modeling study that localized full-length SARS-CoV-2 genomic RNA and relatively small sub-genomic RNA (sgRNA) transcripts across eight subcellular regions, with enrichment, observed in the mitochondrial matrix and nucleolus (Wu et al. 2020). Comparative analysis showed that SARS-CoV-2 RNA exhibited significantly stronger mitochondrial matrix and nucleolar residency signals than SAR-CoV-1 and related coronaviruses (Wu et al. 2020). Notably, it was hypothesized that the presence of SARS-CoV-2 in the mitochondrial matrix was a strong indicator of local translation of sgRNA transcripts and the expression of inhibitory proteins, including the open reading frame ORF9b (Wu et al. 2020). ORF9b was shown to directly interact with mitochondrial TOMM70 to downregulate host cell immunity and facilitate viral replication (Wu et al. 2020). These authors hypothesized that the high degree of similarity between endoplasmic reticulum-derived double-membrane vesicles (DMVs) required for SARS-CoV-2 replication and the double membrane of the mitochondrial matrix might be indicative of a common mechanism of intracellular viral RNA transfer and functional sequestration (Wu et al. 2020). Subsequent reviews have hypothesized a link between SARS-CoV-2 infection and replication that requires successful takeover of mitochondrial processes (Singh et al. 2020; Wang et al. 2020). The findings from several studies that have been reviewed support multiple sites of action of viral proteins expressed by SARS-CoV-2 sgRNA transcripts to suppress essential mitochondrial functions resulting in down-regulation of innate and adaptive immune processes (Singh et al. 2020).

Accordingly, a unified hypothesis of the mechanism of action for functional entrapment, or takeover, of host mitochondria, involves targeted, potentially synergistic, effects of small viral proteins expressed from sgORFs or sgRNA transcripts at critical regulatory sites within the mitochondrial matrix. The scope of potential inhibitory effects mediated by small viral proteins within the mitochondrial matrix may extend to protein complexes composed of subunits expressed from both normal DNA and mtDNA. Whereas the findings of the computational analyses by Wu and coworkers await secondary validation by complementary empirical studies, these observations have implications for advancing the understanding of the systemic harmful effects of SARS-CoV-2 infection in patients with COVID-19. Infection with SARS-CoV-2 is both organ-specific and systemic. The presence of intact mitochondria that contain SARS-CoV-2 RNA and viral proteins may act as infectious units or as complex inflammatory vehicles. The subsequent regulatory roles of intercellular and blood-borne transport and uptake of functional mitochondria and mtDNA may maintain state-dependent levels of proinflammatory effects.

In COVID -19, a paradoxical viral infection of circulating platelets lacking the required angiotensin-converting enzyme-2 (ACE-2) receptor has been reported in severely ill patients presenting with thrombotic complications associated with SARS-CoV-2 infection (Manne et al. 2020). ACE2-independent uptake of fully functional SARS-CoV-2 genomic mRNA in circulating platelets as an alternate mechanism of viral infection appears to be consistent with the predictive existence of circulating blood-borne mitochondria carrying resident full-length genomic RNA sequestered within the mitochondrial matrix (Wu et al. 2020). Patients with COVID-19 who have thrombotic complications of SARS-CoV-2 infection have been reported to transiently express antiphospholipid autoantibodies, including mitochondrial-derived anticardiolipin immunoglobulins (Dotan et al. 2021). This functional observation supports that intact circulating mitochondria with resident biologically active species of SARS-CoV-2 RNA or small viral proteins act as self-contained complex inflammatory vehicles. The multiple effects of heteroplasmic mtDNA copy numbers to regulate normative mitochondrial dynamics may predict the alteration of the residency patterns of SARS-CoV-2 RNA strains and the expressed viral proteins within the mitochondrial matrix. The functional consequences of interactive heritable and acquired mtDNA polymorphisms on the progression of COVID -19 may then be traced to intrinsic differences in the maternal haplotype associated with bidirectional communication of essential mitochondrial processes with mtDNA gene expression.

## Conclusions

This review has presented a critically oriented discussion of potential functional implications of mtDNA heteroplasmy in human health and disease with special reference to its putative role in dysregulation of metabolic and immunological processes associated with rundown of mitochondrial function in COVID-19 neurological disorders. We contend that mtDNA heteroplasmy serves as a dynamic informational reservoir and key regulatory mediator that facilitates bidirectional communication pathways involved in temporal linkage of nuclear and mitochondrial gene expression found in viruses, prokaryotic, and eukaryotic cells. Chronic dysregulation of mitochondrial function leads to the initiation and persistence of diverse pathophysiological states. It may result from the temporal loss of ongoing restorative metabolic and immunological processes inherently linked to the normative cell-specific patterns of mtDNA heteroplasmy.

We have assembled a working mechanistic model proposed for the progression of COVID-19, which may involve extracellular and blood-borne transport and uptake of intact mitochondria with resident biologically active species of SARS-CoV-2 RNA and viral proteins that function as infectious units or complex inflammatory vehicles. The resulting multiple effects of mtDNA heteroplasmy to regulate normative mitochondrial dynamics may alter the patterns of SARS-CoV-2 RNA species and expressed viral proteins within the mitochondrial matrix. This hypothesis supports the need for empirical studies to test the relationships between the mtDNA heteroplasmy and the immune response to SARS-CoV-2 infection, including in the CNS and in the GI tract.

Complex mitochondrial functions from primordial prokaryotic endosymbiont processes may be dependent on mtDNA heteroplasmy as an informational reservoir. Further understanding of mtDNA copy number heteroplasmic distribution may represent a dynamic algorithm for targeting bidirectional communication processes between nuclear and mitochondrial-genomes in SARS-CoV-2 infection. Increased understanding of mtDNA heteroplasmy in the ongoing COVID-19 pandemic involves successful innate and adaptive immunological defense mechanisms dependent on the conformational matching of shared genetic vocabularies. Heteroplasmy of mtDNA provides a molecular substrate for creating novel enhanced immunological selection relevant to SARS-CoV-2 and future emerging pathogenic viruses.

## References

[CR1] Al Amir Dache Z, Otandault A, Tanos R, Pastor B, Meddeb R, Sanchez C, Arena G, Lasorsa L, Bennett A, Grange T, El Messaoudi S, Mazard T, Prevostel C, Thierry AR (2020) Blood contains circulating cell-free respiratory competent mitochondria. FASEB J 34(3):3616–3630. 10.1096/fj.201901917RR 31957088 10.1096/fj.201901917RR

[CR2] Allen JF (2015) Why chloroplasts and mitochondria retain their own genomes and genetic systems: Colocation for redox regulation of gene expression. PNAS 112:10231–10238. 10.1073/pnas.1500012112 26286985 10.1073/pnas.1500012112 PMC4547249

[CR3] Anderson S, Bankier AT, Barrell BG, de Bruijn MH, Coulson AR, Drouin J, Eperon IC, Nierlich DP, Roe BA, Sanger F, Schreier PH, Smith AJ, Staden R, Young IG (1981) Sequence and organization of the human mitochondrial genome. Nature 290(5806):457–465. 10.1038/290457a0 7219534 10.1038/290457a0

[CR4] Angajala A, Lim S, Phillips JB, Kim JH, Yates C, You Z, Tan M (2018) Diverse roles of mitochondria in immune responses: novel insights into immuno-metabolism. Front Immunol 9:1605. 10.3389/fimmu.2018.01605 30050539 10.3389/fimmu.2018.01605 PMC6052888

[CR5] Aryaman J, Bowles C, Jones NS, Johnston IG (2019) Mitochondrial network state scales mtDNA genetic dynamics. Genetics 212(4):1429–1443. 10.1534/genetics.119.302423 31253641 10.1534/genetics.119.302423 PMC6707450

[CR7] Bedarf JR, Hildebrand F, Coelho LP, Sunagawa S, Bahram M, Goeser F, Bork P, Wullner U (2017) Functional implications of microbial and viral gut metagenome changes in early stage L-DOPA-naive Parkinson’s disease patients. Genome Med 9(1):39. 10.1186/s13073-017-0428-y 28449715 10.1186/s13073-017-0428-y PMC5408370

[CR8] Bernier L, York EM, MacVicar BA (2020) Immunometabolism in the brain: how metabolism shapes microglial function. Trends Neurosci 43(11):854–869 32958333 10.1016/j.tins.2020.08.008

[CR9] Bodea LG, Wang Y, Linnartz-Gerlach B, Kopatz J, Sinkkonen L, Musgrove R, Kaoma T, Muller A, Vallar L, Di Monte DA, Balling R, Neumann H (2014) Neurodegeneration by activation of the microglial complement-phagosome pathway. J Neurosci 34(25):8546–8556. 10.1523/JNEUROSCI.5002-13.2014 24948809 10.1523/JNEUROSCI.5002-13.2014 PMC6608212

[CR10] Braak H, Del Tredici K (2017) Neuropathological staging of brain pathology in sporadic Parkinson’s disease: separating the wheat from the chaff. J Parkinson’s Dis 7(S1):S73–S87. 10.3233/JPD-179001 10.3233/JPD-179001 PMC5345633 28282810

[CR11] Braak H, Rub U, Gai WP, Del Tredici K (2003) Idiopathic Parkinson’s disease: possible routes by which vulnerable neuronal types may be subject to neuroinvasion by an unknown pathogen. J Neural Transm (Vienna) 110(5):517–536. 10.1007/s00702-002-0808-2 12721813 10.1007/s00702-002-0808-2

[CR12] Burtscher J, Cappellano G, Omori A, Koshiba T, Millet GP (2020) Mitochondria: in the cross fire of SARS-CoV-2 and immunity. iScience. 10.1016/j.isci.2020.101631 33015593 10.1016/j.isci.2020.101631 PMC7524535

[CR13] Chandel NS (2014) Mitochondria as signaling organelles. BMC Biol 12:34. 10.1186/1741-7007-12-34 24884669 10.1186/1741-7007-12-34 PMC4035690

[CR14] Crunfli F, Carregari VC, Veras F (2020) SARS-CoV-2 infects brain astrocytes of COVID-19 patients and impairs neuronal viability. 10.1101/2020.10.09.20207464

[CR15] Davila AF, Zamorano P (2013) Mitochondria and the evolutionary roots of cancer. Phys Biol 10(2):026008. 10.1088/1478-3975/10/2/026008 23519071 10.1088/1478-3975/10/2/026008

[CR16] Deo P, Chow SH, Han ML, Speir M, Huang C, Schittenhelm RB, Dhital S, Emery J, Li J, Kile BT, Vince JE, Lawlor KE, Naderer T (2020) Mitochondrial dysfunction caused by outer membrane vesicles from Gram-negative bacteria activates intrinsic apoptosis and inflammation. Nat Microbiol 5(11):1418–1427. 10.1038/s41564-020-0773-2 32807891 10.1038/s41564-020-0773-2

[CR17] Devos D, Lebouvier T, Lardeux B, Biraud M, Rouaud T, Pouclet H, Coron E, Bruley des Varannes S, Naveilhan P, Nguyen JM, Neunlist M, Derkinderen P (2013) Colonic inflammation in Parkinson’s disease. Neurobiol Dis 50:42–48. 10.1016/j.nbd.2012.09.007 23017648 10.1016/j.nbd.2012.09.007

[CR18] Diaz Heijtz R, Wang S, Anuar F, Qian Y, Bjorkholm B, Samuelsson A, Hibberd ML, Forssberg H, Pettersson S (2011) Normal gut microbiota modulates brain development and behavior. Proc Natl Acad Sci USA 108(7):3047–3052. 10.1073/pnas.1010529108 21282636 10.1073/pnas.1010529108 PMC3041077

[CR19] Dobbs RJ, Charlett A, Purkiss AG, Dobbs SM, Weller C, Peterson DW (1999) Association of circulating TNF-alpha and IL-6 with ageing and parkinsonism. Acta Neurol Scand 100(1):34–41. 10.1111/j.1600-0404.1999.tb00721.x 10416510 10.1111/j.1600-0404.1999.tb00721.x

[CR20] Dotan A, Muller S, Kanduc D, David P, Halpert G, Shoenfeld Y (2021) The SARS-CoV-2 as an instrumental trigger of autoimmunity. Autoimmun Rev. 10.1016/j.autrev.2021.102792 33610751 10.1016/j.autrev.2021.102792 PMC7892316

[CR21] Esch T, Stefano GB (2002) Proinflammation: a common denominator or initiator of different pathophysiological disease processes. Med Sci Monitor 8(5):1–9 12011758

[CR22] Esch T, Stefano GB, Ptacek R, Kream RM (2020) Emerging roles of blood-borne intact and respiring mitochondria as bidirectional mediators of pro- and anti-inflammatory processes. Med Sci Monit. 10.12659/MSM.924337 10.12659/MSM.924337 PMC7142321 32225126

[CR23] Forsyth CB, Shannon KM, Kordower JH, Voigt RM, Shaikh M, Jaglin JA, Estes JD, Dodiya HB, Keshavarzian A (2011) Increased intestinal permeability correlates with sigmoid mucosa alpha-synuclein staining and endotoxin exposure markers in early Parkinson’s disease. PLoS ONE 6(12):e28032. 10.1371/journal.pone.0028032 22145021 10.1371/journal.pone.0028032 PMC3228722

[CR24] Hayakawa K, Esposito E, Wang X, Terasaki Y, Liu Y, Xing C, Ji X, Lo EH (2016) Transfer of mitochondria from astrocytes to neurons after stroke. Nature 535(7613):551–555 27466127 10.1038/nature18928 PMC4968589

[CR25] He Y, Wu J, Dressman DC, Iacobuzio-Donahue C, Markowitz SD, Velculescu VE, Diaz LA Jr, Kinzler KW, Vogelstein B, Papadopoulos N (2010) Heteroplasmic mitochondrial DNA mutations in normal and tumour cells. Nature 464(7288):610–614. 10.1038/nature08802 20200521 10.1038/nature08802 PMC3176451

[CR26] Hirose M, Kunstner A, Schilf P, Sunderhauf A, Rupp J, Johren O, Schwaninger M, Sina C, Baines JF, Ibrahim SM (2017) Mitochondrial gene polymorphism is associated with gut microbial communities in mice. Sci Rep 7(1):15293. 10.1038/s41598-017-15377-7 29127319 10.1038/s41598-017-15377-7 PMC5681637

[CR27] Hirose M, Kunstner A, Schilf P, Tietjen AK, Johren O, Huebbe P, Rimbach G, Rupp J, Schwaninger M, Busch H, Ibrahim SM (2019) A natural mtDNA polymorphism in complex III is a modifier of healthspan in mice. Int J Mol Sci 20(9):2359. 10.3390/ijms20092359 31085998 10.3390/ijms20092359 PMC6539666

[CR28] Hoitzing H, Gammage PA, Haute LV, Minczuk M, Johnston IG, Jones NS (2019) Energetic costs of cellular and therapeutic control of stochastic mitochondrial DNA populations. PLoS Comput Biol 15(6):e1007023. 10.1371/journal.pcbi.1007023 31242175 10.1371/journal.pcbi.1007023 PMC6615642

[CR29] Jackson MV, Morrison TJ, Doherty DF, McAuley DF, Matthay MA, Kissenpfennig A, O’Kane CM, Krasnodembskaya AD (2016) Mitochondrial transfer via tunneling nanotubes (TNT) is an important mechanism by which mesenchymal stem cells enhance macrophage phagocytosis in the in vitro and in vivo models of ARDS. Stem Cells 34(8):2210–2223. 10.1002/stem.2372 27059413 10.1002/stem.2372 PMC4982045

[CR30] Jacobi FK, Meyer J, Pusch CM, Wissinger B (2001) Quantitation of heteroplasmy in mitochondrial DNA mutations by primer extension using Vent(R)(exo-) DNA polymerase and RFLP analysis. Mutat Res 478(1–2):141–151. 10.1016/s0027-5107(01)00134-8 11406178 10.1016/s0027-5107(01)00134-8

[CR31] Kitani T, Kami D, Matoba S, Gojo S (2014) Internalization of isolated functional mitochondria: involvement of macropinocytosis. J Cell Mol Med 18(8):1694–1703. 10.1111/jcmm.12316 24912369 10.1111/jcmm.12316 PMC4190914

[CR32] Kosikowska U, Biernasiuk A, Korona-Glowniak I, Kiciak S, Tomasiewicz K, Malm A (2016) The association of chronic hepatitis c with respiratory microbiota disturbance on the basis of decreased Haemophilus spp. colonization. Med Sci Monit 22:625–632. 10.12659/msm.895544 10.12659/MSM.895544 PMC4771098 26912163

[CR33] Lebouvier T, Chaumette T, Paillusson S, Duyckaerts C, Bruley des VarannesNeunlistDerkinderen SMP (2009) The second brain and Parkinson’s disease. Eur J Neurosci 30(5):735–741. 10.1111/j.1460-9568.2009.06873.x 19712093 10.1111/j.1460-9568.2009.06873.x

[CR34] Lechuga-Vieco AV, Latorre-Pellicer A, Johnston IG, Prota G, Gileadi U, Justo-Mendez R, Acin-Perez R, Martinez-de-Mena R, Fernandez-Toro JM, Jimenez-Blasco D, Mora A, Nicolas-Avila JA, Santiago DJ, Priori SG, Bolanos JP, Sabio G, Criado LM, Ruiz-Cabello J, Cerundolo V, Jones NS, Enriquez JA (2020) Cell identity and nucleo-mitochondrial genetic context modulate OXPHOS performance and determine somatic heteroplasmy dynamics. Sci Adv 6(31):eaba5345. 10.1126/sciadv.aba5345 10.1126/sciadv.aba5345 PMC7439646 32832682

[CR35] Lobet E, Letesson JJ, Arnould T (2015) Mitochondria: a target for bacteria. Biochem Pharmacol 94(3):173–185. 10.1016/j.bcp.2015.02.007 25707982 10.1016/j.bcp.2015.02.007

[CR36] Lynch MA (2020) Can the emerging field of immunometabolism provide insights into neuroinflammation? Prog Neurobiol 184:101719 31704314 10.1016/j.pneurobio.2019.101719

[CR37] Main BS, Minter MR (2017) Microbial immuno-communication in neurodegenerative diseases. Front Neurosci 11:151. 10.3389/fnins.2017.00151 28386215 10.3389/fnins.2017.00151 PMC5362619

[CR38] Malkki H (2017) Parkinson disease: could gut microbiota influence severity of Parkinson disease? Nat Rev Neurol 13(2):66–67. 10.1038/nrneurol.2016.195 27982042 10.1038/nrneurol.2016.195

[CR39] Manne BK, Denorme F, Middleton EA, Portier I, Rowley JW, Stubben C, Petrey AC, Tolley ND, Guo L, Cody M, Weyrich AS, Yost CC, Rondina MT, Campbell RA (2020) Platelet gene expression and function in patients with COVID-19. Blood 136(11):1317–1329. 10.1182/blood.2020007214 32573711 10.1182/blood.2020007214 PMC7483430

[CR40] Matschke J, Lütgehetmann M, Hagel C, Sperhake JP, Schröder AS, Edler C, Mushumba H, Fitzek A, Allweiss L, Dandri M, Dottermusch M, Heinemann A, Pfefferle S, Schwabenland M, Sumner Magruder D, Bonn S, Prinz M, Gerloff C, Püschel K, Krasemann S, Aepfelbacher M, Glatzel M (2020) Neuropathology of patients with COVID-19 in Germany: a post-mortem case series. Lancet Neurol 19(11):919–929 33031735 10.1016/S1474-4422(20)30308-2 PMC7535629

[CR41] McCoy-Simandle K, Hanna SJ, Cox D (2016) Exosomes and nanotubes: control of immune cell communication. Int J Biochem Cell Biol 71:44–54. 10.1016/j.biocel.2015.12.006 26704468 10.1016/j.biocel.2015.12.006 PMC4720554

[CR42] McCully JD, Levitsky S, Del Nido PJ, Cowan DB (2016) Mitochondrial transplantation for therapeutic use. Clin Transl Med 5(1):16. 10.1186/s40169-016-0095-4 27130633 10.1186/s40169-016-0095-4 PMC4851669

[CR43] Mok BY, de Moraes MH, Zeng J, Bosch DE, Kotrys AV, Raguram A, Hsu F, Radey MC, Peterson SB, Mootha VK, Mougous JD, Liu DR (2020) A bacterial cytidine deaminase toxin enables CRISPR-free mitochondrial base editing. Nature 583(7817):631–637. 10.1038/s41586-020-2477-4 32641830 10.1038/s41586-020-2477-4 PMC7381381

[CR44] Mulak A, Bonaz B (2015) Brain-gut-microbiota axis in Parkinson’s disease. World J Gastroenterol 21(37):10609–10620. 10.3748/wjg.v21.i37.10609 26457021 10.3748/wjg.v21.i37.10609 PMC4588083

[CR45] Müller-Nedebock AC, van der Westhuizen FH, Koks S, Bardien S (2021) Nuclear genes associated with mitochondrial DNA processes as contributors to Parkinson’s disease risk. Mov Disord. 10.1002/mds.28475 33513296 10.1002/mds.28475

[CR46] Nagakawa K, Soyama A, Hidaka M, Adachi T, Ono S, Hara T, Takatsuki M, Eguchi S (2020) Elevated plasma levels of mitochondria-derived damage-associated molecular patterns during liver transplantation: predictors for postoperative multi-organ dysfunction syndrome. Tohoku J Exp Med 250(2):87–93. 10.1620/tjem.250.87 32062616 10.1620/tjem.250.87

[CR48] Pacheu-Grau D, Gomez-Duran A, Iglesias E, Lopez-Gallardo E, Montoya J, Ruiz-Pesini E (2013) Mitochondrial antibiograms in personalized medicine. Hum Mol Genet 22(6):1132–1139. 10.1093/hmg/dds517 23223015 10.1093/hmg/dds517

[CR49] Pollara J, Edwards RW, Lin L, Bendersky VA, Brennan TV (2018) Circulating mitochondria in deceased organ donors are associated with immune activation and early allograft dysfunction. JCI Insight 3(15):e121622. 10.1172/jci.insight.121622 30089724 10.1172/jci.insight.121622 PMC6129133

[CR50] Quiros PM, Mottis A, Auwerx J (2016) Mitonuclear communication in homeostasis and stress. Nat Rev Mol Cell Biol 17(4):213–226. 10.1038/nrm.2016.23 26956194 10.1038/nrm.2016.23

[CR51] Rietdijk CD, Perez-Pardo P, Garssen J, van Wezel RJ, Kraneveld AD (2017) Exploring Braak’s hypothesis of Parkinson’s disease. Front Neurol 8:37. 10.3389/fneur.2017.00037 28243222 10.3389/fneur.2017.00037 PMC5304413

[CR52] Saint-Georges-Chaumet Y, Edeas M (2016) Microbiota-mitochondria inter-talk: consequence for microbiota-host interaction. Pathog Dis 74(1):ftv096. 10.1093/femspd/ftv096 10.1093/femspd/ftv096 26500226

[CR53] Scheperjans F, Aho V, Pereira PA, Koskinen K, Paulin L, Pekkonen E, Haapaniemi E, Kaakkola S, Eerola-Rautio J, Pohja M, Kinnunen E, Murros K, Auvinen P (2015) Gut microbiota are related to Parkinson’s disease and clinical phenotype. Mov Disord 30(3):350–358. 10.1002/mds.26069 25476529 10.1002/mds.26069

[CR54] Schilf P, Künstner A, Olbrich M, Waschina S, Fuchs B, Galuska CE, Braun A, Neuschütz K, Seutter M, Bieber K, Hellberg L, Sina C, Laskay T, Rupp J, Ludwig RJ, Zillikens D, Busch H, Sadik CD, Hirose M, Ibrahim SM (2021) A mitochondrial polymorphism alters immune cell metabolism and protects mice from skin inflammation. Int J Mol Sci 22(3):1006. 10.3390/ijms22031006 33498298 10.3390/ijms22031006 PMC7863969

[CR55] Shenoy S (2020) Coronavirus (Covid-19) sepsis: revisiting mitochondrial dysfunction in pathogenesis, aging, inflammation, and mortality. Inflamm Res 69(11):1077–1085 32767095 10.1007/s00011-020-01389-z PMC7410962

[CR56] Singh KK, Chaubey G, Chen JY, Suravajhala P (2020) Decoding SARS-CoV-2 hijacking of host mitochondria in COVID-19 pathogenesis. Am J Physiol Cell Physiol 319(2):C258–C267. 10.1152/ajpcell.00224.2020 32510973 10.1152/ajpcell.00224.2020 PMC7381712

[CR57] Sonetti D, Ottaviani E, Bianchi F, Rodriquez M, Stefano ML, Scharrer B, Stefano GB (1994) Microglia in invertebrate ganglia. Proc Natl Acad Sci USA 91:9180–9184. 10.1073/pnas.91.19.9180 8090788 10.1073/pnas.91.19.9180 PMC44771

[CR58] Song E, Zhang C, Israelow B, Lu-Culligan A, Prado AV, Skriabine S, Lu P, Weizman OE, Liu F, Dai Y, Szigeti-Buck K, Yasumoto Y, Wang G, Castaldi C, Heltke J, Ng E, Wheeler J, Alfajaro MM, Levavasseur E, Fontes B, Ravindra NG, Van Dijk D, Mane S, Gunel M, Ring A, Kazmi SAJ, Zhang K, Wilen CB, Horvath TL, Plu I, Haik S, Thomas JL, Louvi A, Farhadian SF, Huttner A, Seilhean D, Renier N, Bilguvar K, Iwasaki A (2021) Neuroinvasion of SARS-CoV-2 in human and mouse brain J Exp Med. 218(3):e20202135 10.1084/jem.20202135 PMC7808299 33433624

[CR59] Song X, Hu W, Yu H, Wang H, Zhao Y, Korngold R, Zhao Y (2020) Existence of circulating mitochondria in human and animal peripheral blood. Int J Mol Sci 21(6):2122. 10.3390/ijms21062122 32204530 10.3390/ijms21062122 PMC7139699

[CR61] Stefano GB, Bilfinger TV, Fricchione GL (1994) The immune neuro-link and the macrophage: postcardiotomy delirium HIV-associated dementia and psychiatry. Prog Neurobiol 42(4):475–488. 10.1016/0301-0082(94)90048-5 8090931 10.1016/0301-0082(94)90048-5

[CR62] Stefano GB, Bjenning C, Wang F, Wang N, Kream RM (2017) Mitochondrial heteroplasmy. Adv Exp Med Biol 982:577–594. 10.1007/978-3-319-55330-6_30 28551808 10.1007/978-3-319-55330-6_30

[CR63] Stefano GB, Kream RM (2016) Mitochondrial DNA heteroplasmy in human health and disease. Biomed Rep 4:259–262. 10.3892/br.2016.590 26998260 10.3892/br.2016.590 PMC4774312

[CR64] Stefano GB, Mantione KJ, Capellan L, Casares FM, Challenger S, Ramin R, Samuel JM, Snyder C, Kream RM (2015) Morphine stimulates nitric oxide release in human mitochondria. J Bioenerg Biomembr 47(5):409–417. 10.1007/s10863-015-9626-8 26350413 10.1007/s10863-015-9626-8

[CR65] Stefano GB, Pilonis N, Ptacek R, Raboch J, Vnukova M, Kream RM (2018) Gut, microbiome, and brain regulatory axis: relevance to neurodegenerative and psychiatric disorders. Cell Mol Neurobiol 38:1197–1206. 10.1007/s10571-018-0589-2 29802603 10.1007/s10571-018-0589-2 PMC6061125

[CR66] Stefano ML, Kream RM, Stefano GB (2020) A novel vaccine employing non-replicating rabies virus expressing chimeric SARS-CoV-2 spike protein domains: functional inhibition of viral/nicotinic acetylcholine receptor complexes. Med Sci Monit. 10.12659/MSM.926016. 10.12659/MSM.926016 PMC7278327 32463026

[CR67] Stefano GB, Ptacek R, Ptackova H, Martin A, Kream RM (2021) Selective neuronal mitochondrial targeting in SARS-CoV-2 infection affects cognitive processes to induce 'brain fog' and results in behavioral changes that favor viral survival. Med Sci Monit. 10.12659/MSM.930886. 10.12659/MSM.930886 PMC7845145 33487628

[CR68] Taanman JW (1999) The mitochondrial genome: structure, transcription, translation and replication. Biochim Biophys Acta 1410(2):103–123. 10.1016/s0005-2728(98)00161-3 10076021 10.1016/s0005-2728(98)00161-3

[CR69] Tatsuta T, Scharwey M, Langer T (2014) Mitochondrial lipid trafficking. Trends Cell Biol 24(1):44–52. 10.1016/j.tcb.2013.07.011 24001776 10.1016/j.tcb.2013.07.011

[CR70] Tiku V, Tan MW, Dikic I (2020) Mitochondrial functions in infection and immunity. Trends Cell Biol 30(4):263–275. 10.1016/j.tcb.2020.01.006 32200805 10.1016/j.tcb.2020.01.006 PMC7126537

[CR71] Tremlett H, Bauer KC, Appel-Cresswell S, Finlay BB, Waubant E (2017) The gut microbiome in human neurological disease: a review. Ann Neurol 81(3):369–382. 10.1002/ana.24901 28220542 10.1002/ana.24901

[CR72] Verheijden S, De Schepper S, Boeckxstaens GE (2015) Neuron-macrophage crosstalk in the intestine: a “microglia” perspective. Front Cell Neurosci 9:403. 10.3389/fncel.2015.00403 26528133 10.3389/fncel.2015.00403 PMC4603243

[CR73] Villaran RF, Espinosa-Oliva AM, Sarmiento M, De Pablos RM, Arguelles S, Delgado-Cortes MJ, Sobrino V, Van Rooijen N, Venero JL, Herrera AJ, Cano J, Machado A (2010) Ulcerative colitis exacerbates lipopolysaccharide-induced damage to the nigral dopaminergic system: potential risk factor in Parkinson`s disease. J Neurochem 114(6):1687–1700. 10.1111/j.1471-4159.2010.06879.x 20584104 10.1111/j.1471-4159.2010.06879.x

[CR74] Wang X, Gerdes HH (2015) Transfer of mitochondria via tunneling nanotubes rescues apoptotic PC12 cells. Cell Death Differ 22(7):1181–1191. 10.1038/cdd.2014.211 25571977 10.1038/cdd.2014.211 PMC4572865

[CR75] Wang F, Kream RM, Stefano GB (2020) Long-term respiratory and neurological sequelae of COVID-19. Med Sci Monit 26:e928996. 10.12659/MSM.928996 33177481 10.12659/MSM.928996 PMC7643287

[CR76] West AP, Shadel GS, Ghosh S (2011) Mitochondria in innate immune responses. Nat Rev Immunol 11(6):389–402. 10.1038/nri2975 21597473 10.1038/nri2975 PMC4281487

[CR77] Wu KE, Fazal FM, Parker KR, Zou J, Chang HY (2020) RNA-GPS predicts SARS-CoV-2 RNA residency to host mitochondria and nucleolus. Cell Syst 11(1):102–108e103. 10.1016/j.cels.2020.06.008 10.1016/j.cels.2020.06.008 PMC7305881 32673562

[CR78] Yardeni T, Tanes CE, Bittinger K, Mattei LM, Schaefer PM, Singh LN, Wu GD, Murdock DG, Wallace DC (2019) Host mitochondria influence gut microbiome diversity: a role for ROS. Sci Signal 2(588):eaaw3159. 10.1126/scisignal.aaw3159 10.1126/scisignal.aaw3159 31266851

[CR79] Zhou L, Foster JA (2015) Psychobiotics and the gut-brain axis: in the pursuit of happiness. Neuropsychiatr Dis Treat 11:715–723. 10.2147/NDT.S61997 25834446 10.2147/NDT.S61997 PMC4370913

[CR80] Zhu M, Barbas AS, Lin L, Scheuermann U, Bishawi M, Brennan TV (2018) Mitochondria released by apoptotic cell death initiate innate immune responses. Immunohorizons 2(11):384–397. 10.4049/immunohorizons.1800063 30847435 10.4049/immunohorizons.1800063 PMC6400482

[CR81] Zuo H, Wan Y (2019) Metabolic reprogramming in mitochondria of myeloid cells. Cells 9(1):5. 10.3390/cells9010005 31861356 10.3390/cells9010005 PMC7017304

